# A Novel Device for Assessment and Treatment of Upper Cervical Spine: Test–Retest Reliability Study

**DOI:** 10.3390/jcm12051954

**Published:** 2023-03-01

**Authors:** Andoni Carrasco-Uribarren, Xavier Marimon, Alejandro Portela, Sara Cabanillas-Barea, Pere Ramón Rodríguez-Rubio, Román A. Pérez

**Affiliations:** 1 Faculty of Medicine and Health Sciences, Universitat International de Catalunya, 08195 Barcelona, Spain; 2 Bioengineering Institute of Technology, Universitat Internacional de Catalunya (UIC), 08190 Barcelona, Spain; 3 Automatic Control Department, Universitat Politècnica de Catalunya (UPC-BarcelonaTECH), 08034 Barcelona, Spain

**Keywords:** cervical, spine, strength, biomedical engineering, physiotherapy

## Abstract

Introduction: Neck pain is one of the most frequent reasons for consultation in primary care. Clinicians evaluate different variables, including movement and cervical strength, to determine the prognosis of patients. Usually, the tools employed for this purpose are expensive and bulky, or more than one is needed. This study aims to describe a novel device designed to assess the cervical spine and describe its test–retest reliability. Methods: The Spinetrack device was designed to measure the strength of deep cervical flexor muscles and the chin-in and chin-out movement of the upper cervical spine. A test–retest reliability study was developed. The flexion, extension and strength needed to move the Spinetrack device were registered. Two measurements were developed, with one week between each assessment. Results: Twenty healthy subjects were evaluated. The strength of the deep cervical flexor muscles in the first measurement was 21.18 ± 3.15 Newtons, the displacement movement during chin-in movement was 12.79 mm ± 3.46 and the displacement during chin-out movement was 35.99 mm ± 4.44. The test–retest reliability of strength was ICC 0.97 (95% CI (0.91–0.99)). Conclusion: The Spinetrack device has shown excellent test–retest reliability values for the measurement of the strength of the cervical flexor muscles and for the chin-in and chin-out movements.

## 1. Introduction

Cervical pain is an unpleasant sensation perceived in the neck region that can radiate to the arms and last more than 24 h [1]. It is estimated that between 22% and 70% of the world’s population will experience neck pain at least once in their lifetime [2]. Although most cases of acute neck pain go away with or without treatment, approximately 50% of cases will continue to experience pain in the future [3].

International health organisations, such as the International Federation of Orthopaedic Manipulative Physical Therapists, recommend examining patients before treating them [4]. Patients with neck pain usually show upper cervical spine range of motion (ROM) restriction and deep flexor muscles weakness [2]. The specific assessment of cervical and upper cervical spine ROM and muscles strength is important to classify and control the status of patients [4]. Thus, the quantification of ROM and muscles strength is essential for controlling the evolution of patients who come to physiotherapy with several pathologies and dysfunctions [2]. ROM restriction and deep flex IFOMPT or muscles weakness can lead to neck pain, neck stiffness, dizziness, headache, and other symptoms [5,6].

Upper cervical ROM can be assessed by performing the chin-in/chin-out movement while resting the occiput on a support (e.g., a table or t wall) [7,8]. To measure this movement, the patient rests their occiput on the wall and, while maintaining contact with it, moves their head in a flexion motion (chin in) and an extension motion (chin out) [7,8]. To quantify the movement of the chin-in and chin-out, the CROM device (floating compass; Plastimo Airguide, Inc, The Buffalo Groove, IL, USA) is usually used, but other devices such as inertial sensors [9] and photometry can also be used [10].

The chin-in and chin-out movements are also used to test deep muscle strength. Specifically, the chin-in movement activates the deep flexor muscles and minimises the activation of the superficial muscles, such as the sternocleidomastoids [11]. The deep flexor muscles have been shown to be relevant in maintaining cervical lordosis [12]. The function of these muscles has been evaluated by performing the chin-in movement with the pressure biofeedback unit, a test known as CCFT [13,14].

Originally, the CCFT [13,14] was designed as a clinical indicator of the altered activation of the deep cervical flexor muscles. However, this test has a few shortcomings, including the measurement of force in mmHg [13] instead of Newtons (the universal unit for quantifying force). The test is difficult to perform because there is friction between the patient’s head and the surface on which it rests. Furthermore, the test requires multiple instruments, such as the CROM [7] and pressure biofeedback unit [13,14] to quantify the ROM of upper cervical spine and the strength of deep cervical muscles during the CCFT.

Considering the problems associated with CCFT, a novel device has been developed called Spinetrack. The Spinetrack device makes it easier to perform the chin-in and chin-out movements. With it, the ROM of the upper cervical spine and the strength of the deep cervical flexor muscles can be recorded. The friction between the head and the surface where the occiput is resting decreases because the device incorporates a sliding surface. Furthermore, Spinetrack has a force-resistive pressure sensor to measure muscle strength in newtons. The primary objective of this study was to describe the test–retest reliability of the strength and ROM of the upper cervical spine. The secondary objective was to describe a device designed for the evaluation of deep flexor neck strength and upper cervical spine ROM.

## 2. Materials and Methods

### 2.1. Design

The study is divided into two main sections: (1) a description of the cervical assessment and treatment device (Spinetrack prototype device (Universitat International de Catalunya, Sant Cugat del Vàlles, Spain)) and (2) a pilot study of the test–reliability of the device.

In the first section, the operation of the device is described. The Spinetrack device can be used for the evaluation of strength during the chin-in movement and, in a complementary way, can evaluate displacement during the chin in and chin-out movement.

The second section of the study focuses on the test–retest reliability. The assessment of the strength during the chin-in movement and displacement during the chin-in and chin-out movements were measurede. A pilot study was designed with twenty healthy subjects, and a three-stage reproducibility protocol was followed: the training phase, the agreement phase, and the study phase [15].

The Research Ethics Committee of the Universitat International de Catalunya approved this study (FIS-2022-002). Participants provided informed written consent before being enrolled and were able to withdraw their consent at any time during the study in compliance with WHO standards and the Declaration of Helsinki.

#### 2.1.1. Part 1: Device Specifications and Calibration

**Figure 1:**
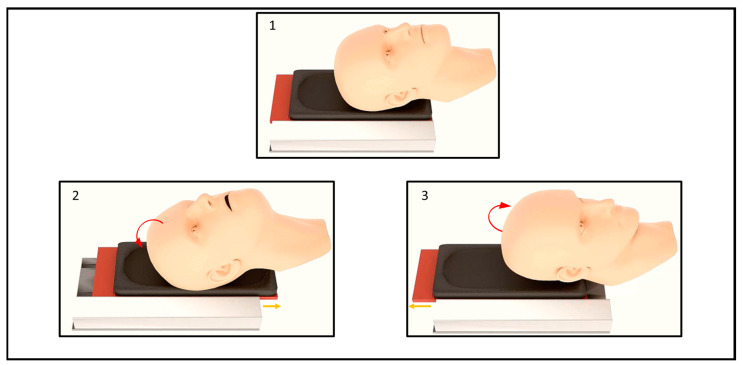
Rendering of the Spinetrack device. The figure shows the movement of the cervical spine performed by the patient on the Spinetrack device and the direction of movement of the mobile platform. (**1**) Cervical rest on the Spinetrack device in neutral position. (**2**) Cervical movement to extension, the mobile platform moves to caudal (**3**) Cervical movement to flexion, the mobile platform moves to cranial.

The main objective of the innovative device is to assess the strength of deep cervical muscle during the chin-in movement. Additionally, the device can objectify the upper cervical movement by the chin-in and chin-out movement. Both assessments can be carried out in a comfortable, fast, safe and reliable way. The Spinetrack device is a sliding platform upon which the subject rests their head and actively performs flexion and extension movements of the upper cervical spine (Figure 1).

##### Description of the Technical System

**Figure 2:**
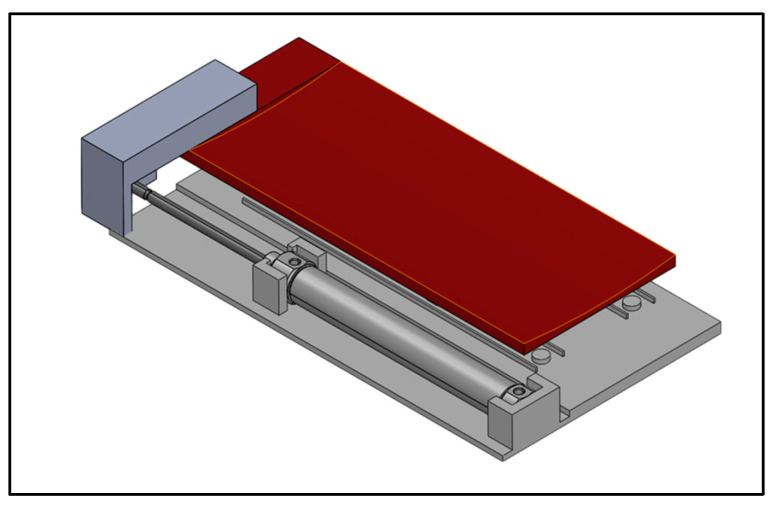
Spinetrack device.

Different models of the Spinetrack device have been developed. A basic model with few sensors, and a more complex model with more sensors and a pneumatic system (Figure 2).

As described, there are different models. The simple version has a built-in high-resolution distance sensor that is coupled to an electronic system that provides information on the distance travelled by the mobile platform with respect to the fixed surface during upper cervical spine flexion and extension.

The extended version of the device has the capability of collecting the same information as the previous one and additionally registers the force needed by the subject to move forward on the mobile surface. A pneumatic system and a force resistive pressure sensor are incorporated into this version. In addition, when the Spinetrack device is used as a training device (training mode), the resistance exerted by the pneumatic system can be regulated through a proportional-integral-derivate (PID) flow controller.

The information obtained by the sensors is processed by a custom-based algorithm (Matlab r2022b, Mathworks, Boston, MA, USA) that applies a moving average filter to reduce acquisition noise and returns the distance travelled by the mobile platform and the force applied to move this platform in SI units, that is, millimetres (mm) and Newtons (N), respectively. Before making any recording, the device needs to be calibrated. For calibration, the Spinetrack device must be connected and held for five seconds in the middle position. Subsequently, it must slide as much as possible towards one end and towards the other, keeping it in each of them for five seconds.

#### 2.1.2. Part 2: Test–Retest Reliability Study

##### Sample and Selection

The sample was recruited from the Universitat International de Catalunya community through advertisements posted in the biomechanical research room and through word of mouth.

Participants were eligible to be included in the study if they were older than 18 years of age and if they did not feel any symptoms at the neck and had not felt any neck symptoms for the past 6 months.

The exclusion criteria were a history of surgery in the spine area, structural pathology such as radiculopathy, disc degeneration, arthritis, osteoarthritis, pregnancy, metabolic diseases, neurological diseases or cracks or fractures in the region of the cervical spine or shoulder [9].

##### Instrumentation and Measures

Prior to testing, the demographic characteristics of the subjects, such as age, gender, weight and height, were recorded. In addition, through the Spinetrack device, the strength of the deep cervical flexor muscles during the chin-in movement and the range of displacement during the chin-in and chin-out movement were measured.

Before data recording, the Spinetrack device was calibrated. After the calibration, the strength of the deep cervical flexor muscles was measured by performing the chin-in movement on the device. The patient rested the head on the device and stayed in supine position. When the test started, the patient was asked to perform the chin-in movement; instead, the device exerted resistance. The force was obtained in Newtons and determined based on the resistance that the patient was able to overcome. Additionally, the maximum painless chin-in and chin-out movement were measured. The patients were asked to perform the maximum painless flexion and extension of the upper cervical spine while resting their heads on the device in the supine position, during this movement the displacement of the mobile surface on the fixed surface occurred and this displacement was registered. The device was connected through a USB port to a laptop, and the data of displacement in mm were extracted (three movements were measured: flexion, extension and full displacement).

##### Testing Procedure

Once the subjects were included in the study, the assessment was conducted in the Laboratory of Biomechanics and Exercise Physiology at the Universitat International de Catalunya.

Prior to the evaluation process, the subjects performed a warm-up during 5 min moving the head in all directions to warm the neck tissues. After the warm-up, the participants laid on their backs with the occiput resting on the device.

Deep flexor muscle strength was the first test, which was obtained from the mean of three strength tests. The examiner A explained to the participants that they had to perform the chin-in movement to assess the strength of the deep cervical flexor muscles. The device was placed in force assessment mode. It was explained to the subjects that they had to perform the maximum chin-in movement without detaching the occiput from the device, if the examiner detected the activation of the sternocleidomastoid, the test was stopped, and the data recorded. To facilitate the movement, the subjects started in a standardized chin-out position, looking down, with the mouth slightly open and the tongue in contact with the upper teeth. Each strength test lasted approximately 5 s, where the patient had to increase the force progressively until reaching maximum strength at the end of the test, moving the head from the chin-out position to the chin-in position. The examiner A encouraged the subjects to reach the maximum voluntary contraction possible. This assessment is similar to other assessment protocols described in the literature [16,17,18].

To assess the displacement of the mobile surface on the fixed surface the participants had to perform the chin-in and chin-out movement. The patients were asked to perform flexion and extension of the upper cervical spine, trying not to detach the occipital area from the device, the movements always were performed in the same order, first toward flexion and then toward extension. During the movement, a displacement of the mobile surface occurs over the fixed surface. The maximum movement for flexion and extension was made three times, each movement was measured and the average of the three movements was calculated. The full displacement was also calculated by adding flexion and extension movements [19].

**Figure 3:**
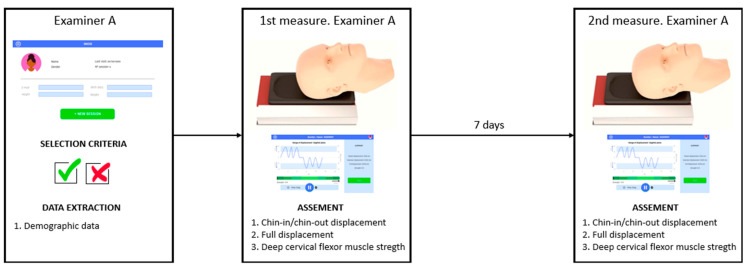
Flowchart of the study protocol.

These evaluations were performed by a physical therapist with more than 15 years of clinical experience. The assessments were performed at two different times—the first was after the subjects were included in the study, and the second was one week after the first evaluation. This type of protocol is commonly used in validity and test–retest reliability studies and has been carried out several times to estimate the test–retest intra- and inter-rater reliability of the measurement of deep cervical flexor muscles strength [20,21] (Figure 3).

##### Data Analysis

All statistical analyses were performed using SPSS Statistics version 22 (IBM, New York, NY, USA). Descriptive statistics were used to characterize the sample. Test–retest reliability statistics between sessions (muscle tests and displacement tests) were computed. An intraclass correlation coefficient (ICC; two-way random, absolute agreement) and respective 95% confidence interval were used for relative test–retest reliability analyses and interpret as poor (ICC < 0.50), moderate (ICC = 0.50–0.75), good (CCI = 0.75–0.90) and excellent (CCI ≥ 0.90).

## 3. Results

20 healthy participants were included at the study (14 females and 6 males; mean age ± standard deviation 31 years ± 13.76), with a weight of 70 kg ± 13.05, and a height of 171.50 cm ± 9.25 were included in the study.

During the first evaluation session, the mean displacement mobility obtained for flexion was 12.79 mm ± 3.46, towards extension was 35.99 mm ± 4.44, and the total range of displacement of 48.79 mm ± 5.99 were found. The mean strength shown by the deep cervical flexor muscles is 21.18 N ± 3.15. These data and the data of the second day of measurement were described in Table 1.

The evaluation protocols described with the novel device have shown excellent test–retest reliability for each of the evaluations carried out, both for the evaluation of the displacements of the device during the movements of the upper cervical spine and for the evaluation of the strength of the deep cervical flexor muscles (Table 2).

## 4. Discussion

International organisations, such as the International Federation of Orthopaedic Manipulative Physical Therapists, recommend carrying out objective assessments of any neuromusculoskeletal dysfunction prior to any treatment. Clinically it is necessary to develop instruments that allow evaluating patients objectively to know the state in which they are and how they are evolving. The present study aims to present a novel device that allows for an evaluation of the strength of the deep cervical flexor muscles in Newtons and to evaluate the range of displacement during the chin-in and chin-out movement. In addition, the first data obtained from the evaluation of twenty healthy subjects are presented showed an excellent as are the test–retest reliability ratings between two evaluations carried out with a week of rest between evaluations.

The assessment of muscle strength is important because it comprises the reference parameters used when the exercise is prescribed and with which it is compared at follow-up. Additionally, muscle strength or endurance assessments are part of the physical examination, and their respective results are then used to classify patients [22]. There are different methods to measure the strength and resistance of the cervical musculature, such as hand dynamometer, isometric and isokinetic dynamometer, inflatable pressure biofeedback unit and other measures of muscular performance. Although many instruments and tests are available for cervical assessment, most take up too much space or maybe too expensive for clinical practice. The novel device presented should be affordable for all clinicians and has also been designed with the aim of being small and not taking up too much space in the practice. The design allows it to be easily transported so that training and self-treatment can be carried out at home or elsewhere. However, to achieve this milestone, the device should be more plug and play. In addition, the mobile surface makes easier to movement of chin-in, because the friction is reduced by the sliding surface comparing the friction that the patient has during the same movement in the craneocervical flexion test.

Regarding the data extracted from the device, the strength of the deep cervical flexor muscles ranged from 21.18 N ± 3.15 to 20.78 N ± 3.23 between sessions. It is difficult to compare the data with other studies because a novel testing procedure has been used to develop the assessment. A study by O’Leary et al. [23] showed a novel dynamometer to assess the deep cervical flexor muscles with the chin-in movement. The maximum force they found in healthy subjects was 68.65 N ± 43.15. The differences in strength between both studies may have been caused by the position from which the test was started, O’Leary et al. [23] started in 10° of extension and in the test described in this study the starting position was in full extension. There is a great difference between the results of their study and those in the present study; however, the standard deviation of O’Leary et al.’s [23] work was 62%, unlike the standard deviation of the present study, which was 15%. This demonstrates the greater consistency and validity of our data. The test–retest reliability showed excellent results for the evaluation of the deep cervical flexor muscles: 0.98 (0.91–0.99). These data should be taken with caution because they have been extracted via the evaluation of 20 healthy subjects, with an adapted evaluation protocol and only carried out by a single examiner who was involved with the project; nonetheless, the test–retest reliability is higher than that shown by other studies, which ranges from 0.82 (0.67–0.91) to 0.86 (0.77–0.93) [20].

To measure the ROM of upper cervical spine the chin-in and chin-out movement is used. Usually, the CROM device is used to objectify the ROM of the upper cervical spine but other methods as the photometry and inertial measurements unit have been used for the purpose. Currently, the Spinetrecak device is designed to measure the ROM during the chin-in and chin-out movements by the displacement occurred between the mobile and fixed surface, the measurements unit used are the mm. In addition, the Spinetrack device allows us to extract the full displacement in this plane of movement. The total displacement found in this study was 48.79 mm ± 5.99 for the first session and 47.23 mm ± 7.12 for the second. Test—retest reliability ranged from good to excellent. These values are analogous to those found in a study by Pérez-Fernández T. et al. [9] although in his case, they only assessed the chin-in movement during the craneocervical flexion test, unlike the present study, which also assessed the displacement during the movement of extension.

During the pilot study, some limitations were noted. The Spinetrack device prototype was used for the pilot study, and on three occasions, the device slipped on the treatment table, making measurements difficult and affecting the results. The study was only carried out with healthy subjects, without calculating the sample size, and with a single examiner; the study must be carried out with subjects with neck pain and with an inexperienced evaluator. However, although the physiotherapist who performed the assessment was a clinician with more than 15 years of experience, it was the first time that this physiotherapist had used the device, a fact that strengthens the results obtained. The other limitation is the difficulty of comparing the data with other studies, the units of measurement in which the device registers the movement, and the force are units that are not frequently used for the upper cervical spine. Finally, it must be mentioned that to maintain the industrial secret of the patent, the person who carried out the evaluations was a person involved in it; this could have contributed to better results. As a prospective, we believe that with a slight modification the Spinetrack device could measure the force of the extensor musculature.

## 5. Conclusions

The Spinetrack device has shown an excellent test–retest reliability for measuring of the deep cervical flexor muscles strength and the displacement during the chin-in movement. This novel device has shown a good test–retest reliability for measuring the total displacement and for the displacement that occurred during extension. The results obtained are promising but more studies evaluating the validity and reliability of this device are needed. The results must be taken with caution because the test–retest reliability was calculated with a single examiner who was involved project; changing this could have contributed to better results.

## 6. Patents

This manuscript describes and shows the reliability data of the use of Spinetrack device patent, with application number in Spain of 21-0575 and PCT application number ES2022/070644.

## Figures and Tables

**Table 1:** Descriptive data of displacement movement and strength of deep cervical flexor muscles.

	1st Measure Mean ± SD	2nd Measure Mean ± SD
Strength DCF muscle (N)	21.18 ± 3.15	20.78 ± 3.23
Chin-in displacement (mm)	12.79 ± 3.46	13.04 ± 2.56
Chin-out displacement (mm)	35.99 ± 4.44	35.19 ± 3.92
Full displacement (mm)	48.79 ± 5.99	47.23 ± 7.12

DCF, deep cervical flexor.

**Table 2:** Reliability of displacement movement and strength of deep cervical flexor muscles.

Test—Retest Reliability	ICC (95%)
Strength DCF muscles (N)	0.98 (0.91–0.99)
Chin-in displacement (mm)	0.92 (0.63–0.98)
Chin-out displacement (mm)	0.89 (0.58–0.97)
Full displacement (mm)	0.86 (0.43–0.96)

DCF, Deep cervical flexor.

## Data Availability

The data analysed in this study are included in this published article. The dataset is available from the corresponding author on reasonable request.

## References

[1] Safiri S., Kolahi A.A., Hoy D., Buchbinder R., Mansournia M.A., Bettampadi D., Ashrafi-Asgarabad A., Almasi-Hashiani A., Almasi-Hashiani A., Sepidarkish M. (2020). Global, regional, and national burden of neck pain in the general population, 1990-2017: Systematic analysis of the Global Burden of Disease Study 2017. BMJ.

[2] Blanpied P.R., Gross A.R., Elliott J.M., Devaney L.L., Clewley D., Walton D.M., Sparks C., Robertson E.K. (2017). Neck Pain: Revision 2017. J. Orthop. Sport. Phys. Ther. Mov. Sci. Media.

[3] Cohen S.P. (2015). Epidemiology, diagnosis, and treatment of neck pain. Mayo Clin. Proc..

[4] Rushton A., Carlesso L.C., Flynn T., Hing W.A., Rubinstein R., Vogel S.M., Kerry R. (2023). International Framework for Examination of the Cervical Region for potential of vascular pathologies of the neck prior to Orthopaedic Manual Therapy (OMT) Intervention: International IFOMPT Cervical Framework. J. Orthop. Sport. Phys. Ther..

[5] Carrasco-Uribarren A., Rodriguez-Sanz J., López-de-Celis C., Pérez-Guillen S., Tricás-Moreno J.M., Cabanillas-Barea S. (2021). Short-term effects of the traction-manipulation protocol in dizziness intensity and disability in cervicogenic dizziness: A randomized controlled trial. Disabil. Rehabil..

[6] González-Rueda V., López-de-Celis C., Bueno-Gracia E., Rodríguez-Sanz J., Pérez-Bellmunt A., Barra-López M.E., Hidalgo García C. (2020). Short- and mid-term effects of adding upper cervical manual therapy to a conventional physical therapy program in patients with chronic mechanical neck pain. Randomized controlled clinical trial. Clin. Rehabil..

[7] Ernst M.J., Crawford R.J., Schelldorfer S., Rausch-Osthoff A.K., Barbero M., Kool J., Bauer C.M. (2015). Extension and flexion in the upper cervical spine in neck pain patients. Man. Ther..

[8] Dhimitri K., Brodeur S., Croteau M., Richard S., Seymour C.J. (2013). Reliability of the Cervical Range of Motion Device in Measuring Upper Cervical Motion. J. Man. Manip. Ther..

[9] Pérez-Fernández T., Armijo-Olivo S., Liébana S., Ortíz P.J.D.L.T., Fernández-Carnero J., Raya R., Martín-Pintado-Zugasti A. (2020). A novel use of inertial sensors to measure the craniocervical flexion range of motion associated to the craniocervical flexion test: An observational study. J. Neuroeng. Rehabil..

[10] Falla D.L., Campbell C.D., Fagan A.E., Thompson D.C., Jull G.A. (2003). Relationship between cranio-cervical flexion range of motion and pressure change during the cranio-cervical flexion test. Man. Ther..

[11] Falla D., Jull G., Dalvalba P., Rainoldi A., Merletti R. (2003). An Electromyographic Analysis of the Deep Cervical Flexor Muscles in Performance of Craniocervical Flexion. Phys. Ther..

[12] Mayoux-Benhamou M.A., Revel M., Vallée C., Roudier R., Barbet J.P., Bargy F. (1994). Longus colli has a postural function on cervical curvature. Surg. Radiol. Anat..

[13] Jull G.A., O’Leary S.P., Falla D.L. (2008). Clinical Assessment of the Deep Cervical Flexor Muscles: The Craniocervical Flexion Test. J. Manip. Physiol. Ther..

[14] Jull G., Barrett C., Magee R., Ho P. (1999). Further clinical clarification of the muscle dysfunction in cervical headache. Cephalalgia.

[15] Patijn J. (2019). Reproducibility protocol for diagnostic procedures in Manual/Musculoskeletal Medicine: Edition 2019. Man. Medizin..

[16] O’Leary S., Falla D., Jull G. (2011). The relationship between superficial muscle activity during the cranio-cervical flexion test and clinical features in patients with chronic neck pain. Man. Ther..

[17] O’Leary S., Falla D., Jull G., Vicenzino B. (2007). Muscle specificity in tests of cervical flexor muscle performance. J. Electromyogr. Kinesiol..

[18] Falla D., Jull G., O’Leary S., Dall’Alba P. (2006). Further evaluation of an EMG technique for assessment of the deep cervical flexor muscles. J. Electromyogr. Kinesiol..

[19] Amiri M., Jull G., Bullock-Saxton J. (2003). Measurement of Upper Cervical Flexion and Extension with the 3-Space Fastrak Measurement System: A Repeatability Study. J. Man. Manip. Ther..

[20] Jørgensen R., Ris I., Falla D., Juul-Kristensen B. (2014). Reliability, construct and discriminative validity of clinical testing in subjects with and without chronic neck pain. BMC Musculoskelet. Disord..

[21] Portney L.G., Watkins M.P., Davis F.A. (2009). Foundations of Clinical Research: Applications to Practice.

[22] Villanueva-Ruiz I., Falla D., Lascurain-Aguirrebeña I. (2022). Effectiveness of Specific Neck Exercise for Nonspecific Neck Pain; Usefulness of Strategies for Patient Selection and Tailored Exercise-A Systematic Review with Meta-Analysis. Phys. Ther..

[23] O’Leary S., Vicenzino B., Jull G. (2005). A new method of isometric dynamometry for the craniocervical flexor muscles. Phys. Ther..

